# Supplementary material for: Do extraordinary science and technology scientists balance their publishing and patenting activities?
Source: PLoS One. 2021 Nov 4;16(11):e0259453. doi: 10.1371/journal.pone.0259453 (PMC8568124; doi:10.1371/journal.pone.0259453)
Supplement: Attachment — Submitted filename: 04-reviewers-comments_plos-one.docx [file pone.0259453.s001.docx]

**Responses to Reviewers’ Comments**

Thank you for the constructive comments on our manuscript. We have revised the paper based on the reviewer’ comments and recommendations. Revisions of the manuscript are highlighted in yellow. In addition, the point-by-point responses to the reviewer comments are appended below.

**Reviewer #1:**

1. **First, I suggest the authors to better specify the focus of the paper and how it is related to the research questions. I was puzzled by the fact that the study mentions many different issues, including the relationship between science, technology, and academia (rows 15-27), the bibliometric and patenting profiles of outstanding scientists (rows 39-40), and if productivity, citation, and patent metrics are reliable indicators of scientific/technological excellence (rows 158-167), In my opinion, the main result of the paper is that these indicators are not reliable, as the outstanding scientists examined display highly diversified citation and patent profiles (rows 475-477, 531-532, and 552-553). I see that the two other topics are somehow related to this result, but I think that a more concise and clear presentation of the real focus of the paper would help the reader to follow the argument. In particular, I suggest to better organize the Introduction and the Discussion and Conclusion sections, as some paragraphs seem to be scarcely connected with the main argument. For instance, the paragraph on Nobel laureates (rows 28-38) interrupts the presentation of the rationale of the study and it seems to me not relevant for the discussion, at least at this point of the paper. Similarly, the paragraph on the evaluation of societal effects (rows 520-530) seems to me scarcely related to the focus of the study.**

Response:

Thank you for your constructive suggestions and comments. We have reorganized and revised the Introduction and the Discussion and Conclusions sections. In the Introduction section, we have revised the third research question and have deleted disconnected sentences including original lines 15–40 to clarify and strengthen statements and arguments. Regarding the Discussion and Conclusions section, in addition to deleting the irrelevant statements that you mentioned, we have added new references and made considerable revisions to improve and clarify the statements related to the discussion and conclusion.

2. **Fig. 1: In the Lauterbur facet there is a blue line, does it mean something? If this is the case, please add the explanation.**

Response:

We have added an explanation for the blue vertical line in two scientists’ data to the description of Fig. 1. The blue vertical line indicated the year when the scientist was awarded the Nobel Prize.

3. **Fig. 4 should be explained more extensively: what does the color code mean, for instance? What is “n” in the last row on the left? I suggest the author at least to expand the caption of this figure.**

Response:

We have revised Fig 4 to enhance the visual effect and reader comprehension. More details on Fig 4 have been added to the corresponding paragraph. In addition, we have expanded the caption of Fig 4.

4. **Fig. 5: it is not clear the rationale for the three types of scientists. Why were these ranges chosen to delimit type-B from type-C scientists? I suggest also to indicate the type by a color to improve the readability of the figure.**

Response:

We have added statements to clarify the rationale for three types of scientists. We have also revised Fig 5 to improve its visual effect, including the use of colors for scientists of different types.

5. **The authors cited mostly the appropriate literature, even if some key references that would perfectly fit in their argument are lacking. In particular, the sections on the relationship between citations and research quality should be integrated with a discussion of Aksnes et al. (2019) (DOI: 10.1177/2158244019829575) and similar contributions. Other references are suggested in the attached list of minor comments.**

Response:

We have added the three articles that you suggested and other references to the list of references and incorporated them into the statements and discussions related to citations, research quality, influence, and contributions.

6. **Lastly, I would suggest the authors get editing help from someone with full professional proficiency in English, as the meaning of some phrases and sentences in the manuscript is not always clear (see the list of minor comments attached).**

Response:

The manuscript has undergone professional editing.

7. **Minor revisions and typos:**

**Row 19. “Notably” does not seem to me the right word. Maybe “However”?**

Response:

We have replaced “Notably” with “However.”

**Rows 28-38: I do not understand why this paragraph on Nobel laureates is placed here. It interrupts the argument. I would remove it.**

Response:

We have deleted this paragraph.

**Row 42: “in sample scientists”**

Response:

We have deleted the word “sample.”

**Row 59: I am not sure that “assumption” is the right word here. Maybe “hypothesis” is better?**

Response:

We have replaced “assumption” with “hypothesis.”

**Row 89: Not sure “demonstrated” is the right word here. I suggest the authors to rephrase the entire sentence.**

Response:

We have revised the third research question.

**Row 139-140: The meaning of this sentence is not clear. I suggest the authors to rephrase it.**

Response:

We have rephrased this sentence.

**Rows 143-145: I suggest the authors to integrate the discussion by commenting on Aksnes et al. (2019) (10.1177/2158244019829575), that is an in-depth review of the relationship between research quality and citations.**

**Row 162: I suggest the authors to add reference to Baccini and De Nicolao (2016) (10.1007/s11192-016-1929-y), a relevant study for this debate.**

**Ref. 32 I think it is better to cite e.g., MacRoberts and MacRoberts (2018) (10.1002/asi.23970)**

Response:

We have added the three references as suggested and integrated them into the Literature Review and the Discussion and Conclusions sections.

**Row 209: I am not sure that the word “intelligence” is right here.**

Response:

We have replaced “intelligence” with “output.”

**Row 229: “until” should be replaced with “before”, if a get the meaning of the sentence right.**

Response:

We have replaced “until” with “before.”

**Rows 231-232: It is not very clear how citations were calculated. Is it from the difference between the two?**

Response:

We have rephrased this sentence to enhance clarity.

**Rows 287-288: Accumulation of prizes may be explained also by the Matthew Effect. I suggest the authors to briefly mention this alternative explanation.**

Response:

The relevance of the Matthew effect to our study has been addressed in the Discussion section.

**Row 35: laureates_and**

**Row 127: “one scientific publication of a patent” should be “one scientific publication or a patent”.**

**Row 351: “he had been for a”**

**Ref 25 “orreinforcement” should be replaced with “or reinforcement”**

Response:

We have examined the whole paper and corrected all typos.

**Row 483: I would replace “originate from” with “be reflected in” or “result in”.**

Response:

We have replaced “originate from” with “be reflected in.”

**Rows 485-490: The meaning of this paragraph is not clear to me. I suggest the authors to rephrase it and elaborate a bit on the relationship between inventions and discoveries to clarify the reasoning presented in this paragraph.**

Response:

We have rephrased the relevant text in the Discussion and Conclusions section.

**Ref n. 1: “National inVocation systems” should be changed in “National innovation systems”**

Response:

We have deleted this reference.

**Reviewer #2:**

1. **First, the aims of the paper are unclear. The introduction (p. 4) spells out two very narrow and specific research questions (if these 12 people authored the same number of articles and patents, and if they are highly cited), and a very generic one (if their contributions are "demonstrated" by bibliometric indicators). The authors do not attempt to motivate why these questions are relevant. Then, the analysis only answers to the first two questions (see my point 2 concerning the third question), and the conclusions depart from all three questions, stating that the authors "have demonstrated that bibliometrics indicators are not a reliable measure of the value of discoveries in improving humanity" (pp. 23-24).**

Response:

Thank you for your comments and suggestions. We have rephrased and reorganized the Introduction section to clarify the purpose and research questions of this paper. In particular, we have revised the third research questions and strengthened statements related to the three research questions to enhance the relevance of these statements. In addition, we have revised the Discussion and Conclusions section to ensure the discussions are connected to the research questions.

2. **Second, the paper suffers from confusion in the use of terms and concepts. In some cases, this could be a language problem that the authors could possibly solve by asking a native speaker to proofread the manuscript. This is how I personally (and I myself am not a native speaker) interpret some odd statements such as "The research papers and patents produced ... served as proxies of scientific and technological intelligence" (pp. 9-10) or "The low number of winners indicates that this select group has been responsible for extraordinary contributions" (p. 3). Other examples could be made, but my point is that quite obviously this is not just a language issue.**

**Throughout the paper the authors use "contribution" and "influence" interchangeably (for example in pages 1, and 2), then clearly express the fact that they are not synonymous, but then e.g. on pag. 11 they write again that "citation counts were employed to represent [both] influence and contribution over time". Then for example on pag. 23 they deny this again ("Influence is frequently confused with contribution. In this study, only citation-based counts were used to measure and present scientific and technological influence"). To add to the confusion, sometimes they further add the notions of "performance" and "research originality" (beside the already recalled "intelligence", which I believe to be just a mistake). Perhaps, if the authors clarified better what they aim to measure or document, the statistical analysis too could be more in-depth and precise (see point below).**

Response:

We have used an English editing service to improve our writing quality. Regarding the meaning of some concepts, such as influence, contribution, performance, and research originality, we have examined the full paper and revised related statements. In particular, we have strengthened the rationale for and what we analyzed through our research questions.

3. **Third, the data analysis is rather superficial, and not geared at answering the third (larger) research question. For example, because of variability of citations across a same author's papers, the authors only consider the most highly cited paper for each author.**

Response:

We have revised the third research questions and enhanced the connection between the three research questions.

4. **Further, for years before 1970 the use citation data from Web of Science, and after 1970 from Scopus, without any consideration about how this might affect their results.**

Response:

We have provided further information regarding the citation data collected from Web of Science (WoS) and Scopus. Scopus covers more journals than does WoS. This helped us collect the maximum number of articles published by the 12 scientists. However, Scopus does not provide data on the annual number of citations received by articles before 1970. Thus, for articles published before 1970 and that have received citations before 1970, their total number of citations would be under-counted. To address this problem, we verified whether articles published before 1970 were indexed by WoS and had annual citation count data before 1970. Therefore, for articles published before 1970, their annual citation counts were collected from WoS (before 1970) and Scopus (after 1970).

5. **The authors do not distinguish between citations received before and after the official recognition of the award(s) and therefore cannot tell us if impact (what they call "influence") is due to visibility and/or how is it affected by it.**

Response:

We have recorded citation counts received by year and illustrated the data in Fig 3. In addition, Fig 1 presents the years when each scientist received awards. With reference to Figs 1 and 3, we can observe the changes in citation counts before and after awards. Statements related to citation counts and awards have been added to the Discussion and Conclusions section.

6. **More in general, I do not have precise ideas about how to demonstrate if "contribution" is measured by citation counts, so I am sorry I cannot offer constructive criticisms, but certainly the current analysis does not attempt to answer this question at all. Probably, totally different data would be required (including some measurable evidence of "contribution") and I would personally suggest to the authors to rather change the research question.**

Response:

We have expanded and clarified statements related to citation counts, influence, and contribution as well as our research motives, and we have revised the third research question. As mentioned in our responses to questions 1–3, the description of what we measured for each research question has been expanded and clarified.

7. **Concerning the empirical part, however, my biggest concern is that the authors draw general implications from a very (small and) select sample: both geographically (only US authors) and in terms of impact. They only write (p. 8) that this is an "appropriate" sample for a study of this kind, without much more reflection on this crucial methodological choice.**

Response:

Regarding your biggest concern, we have explained why only 12 subjects were targeted in the study in our Methodology section. To identify scientists with substantial contributions to science and technology, we considered those that had received two specific distinguished awards, with one award emphasizing scientific contribution and the other focusing on technological contribution. We have expanded and clarified our rationale and method used for targeting the 12 scientists.
