# Supplementary material for: Do extraordinary science and technology scientists balance their publishing and patenting activities?
Source: PLoS One. 2021 Nov 4;16(11):e0259453. doi: 10.1371/journal.pone.0259453 (PMC8568124; doi:10.1371/journal.pone.0259453)
Supplement: Attachment — Submitted filename: 03-Response-to-reviewers-comments_plos-one_r2.docx [file pone.0259453.s002.docx]

**Responses to Reviewers’ Comments**

We appreciate the time and efforts that the reviewers have dedicated to providing valuable feedback on our manuscript. We have revised the paper based on the reviewers’ comments. The revisions are highlighted in yellow in the manuscript. Here is a point-by-point response to the reviewers’ comments and concerns.

Reviewer #1:

**Comment 1:** *The new version of the manuscript is clearer and better organized compared to the first one. The paper’s focus is now clearly explained, and the research questions plainly spelled out. However, I still have some troubles with the Introduction, which, in its present form, is too long, with paragraphs that are not always mutually well connected. My suggestion to the authors is to shorten it and remove several details that are already provided in the following sections of the papers (e.g., the debate on the reliability of citation counts for measuring influence is better presented in the Literature Review section, the history of the NMS and NMTI can be left to the Methodology section without weighing the Introduction down, and so on). I would suggest the authors to limit the Introduction to the presentation of the rationale and relevance of their research questions, leaving the context and other details to the Literature Review section.*

**Response:** We agree with this comment, and we have shortened the Introduction section by moving some statements to other sections, leaving the Introduction section clearer and more precise.

**Comment 2:** *Some repetitions may also be reduced to enhance readability throughout the paper (e.g., the fact that filing patents is more challenging than publishing articles is repeated at least three times in different points of the paper).*

**Response:** We have deleted the repetitive statements that you pointed out. And we have examined the whole paper and deleted some other repetitions to enhance readability, thanks for your suggestion.

**Comment 3:** *There are still few typos that should be corrected:* *Abstract: row 30: there is a missing “s” after “scientist”.* *Row 86: it seems the verb is missing in this sentence.* *Row 249: “scientist” should be turned to plural.*

**Response:** We have revised the typos that you pointed out and have also checked the whole paper to correct the other typos.

Reviewer #2:

**Comment 1:** *The authors addressed the most important concerns I and the other referees had raised. There remains a difference in points of views, which should not prevent publication of the manuscript, of course. A final, minor remark: please clarify and streamline throughout the date(s) at which the data are updated. It seems to me, 2018 (for sample selection) and 2019 (for citations data), but this should be more clearly reported.* *Please note that on pag. 11 (row 252) you write "as of 2004": what does that mean, is that a typo?*

**Response:** We have revised the typo that you identified on page 11, which is supposed to be “as of 2019”, thank you for pointing this out. We have also examined all the dates in this paper and made sure that they are correct.

We have indicated that the latest year of the published papers and patents that we used for analysis is 2018. The bibliographic records of the papers, which include the number of citations for each paper, were downloaded on January 29, 2019. Because the cumulative number of citations received by each paper was updated on a date close to the end of 2018, the number of citations received by each paper closely reflects their influence by the end of 2018.

For the patents, we have also elaborated on the relevant statements to clarify the dates. The bibliographic records of the patents were collected in February 2019. Because the number of patent-related citations is not provided in the USPTO database, we calculated the number of citations received on the basis of the references for all patents. The list of references does not change after the patents are granted. Therefore, 2018 is the latest year when the number of patent citations is recorded.
